# Neoadjuvant Chemotherapy in Locally Advanced and Borderline Resectable Nonsquamous Sinonasal Tumors (Esthesioneuroblastoma and Sinonasal Tumor with Neuroendocrine Differentiation)

**DOI:** 10.1155/2016/6923730

**Published:** 2016-02-03

**Authors:** Vijay M. Patil, Amit Joshi, Vanita Noronha, Vibhor Sharma, Saurabh Zanwar, Sachin Dhumal, Shubhada Kane, Prathamesh Pai, Anil D'Cruz, Pankaj Chaturvedi, Atanu Bhattacharjee, Kumar Prabhash

**Affiliations:** ^1^Department of Medical Oncology, Tata Memorial Hospital, Mumbai, India; ^2^Department of Pathology, Tata Memorial Hospital, Mumbai, India; ^3^Department of Surgical oncology, Tata Memorial Hospital, Mumbai, India; ^4^Division of Clinical Research and Biostatistics, Malabar Cancer Centre, Kerala, India

## Abstract

*Introduction.* Sinonasal tumors are chemotherapy responsive which frequently present in advanced stages making NACT a promising option for improving resection and local control in borderline resectable and locally advanced tumours. Here we reviewed the results of 25 such cases treated with NACT.* Materials and Methods*. Sinonasal tumor patients treated with NACT were selected for this analysis. These patients received NACT with platinum and etoposide for 2 cycles. Patients who responded and were amenable for gross total resection underwent surgical resection and adjuvant CTRT. Those who responded but were not amenable for resection received radical CTRT. Patients who progressed on NACT received either radical CTRT or palliative radiotherapy.* Results*. The median age of the cohort was 42 years (IQR 37–47 years). Grades 3-4 toxicity with NACT were seen in 19 patients (76%). The response rate to NACT was 80%. Post-NACT surgery was done in 12 (48%) patients and radical chemoradiation in 9 (36%) patients. The 2-year progression free survival and overall survival were 75% and 78.5%, respectively.* Conclusion*. NACT in sinonasal tumours has a response rate of 80%. The protocol of NACT followed by local treatment is associated with improvement in outcomes as compared to our historical cohort.

## 1. Introduction

Sinonasal tumors are a rare entity [1, 2]. These tumors are usually not included in major head and neck cancer studies addressing questions regarding local management or systemic treatment [3–6]. Hence there is dearth of level 1 evidence in these tumors. Multiple small retrospective series have been published and certain facts are clear from these studies:

1. The subclassification of sinonasal tumors into esthesioneuroblastoma, sinonasal tumor with neuroendocrine differentiation, and sinonasal tumor with poor differentiation helps as the outcome differs according to the exact subtype [7].
2. In all these three subtypes surgical resection with or without adjuvant radiation remains the cornerstone of management [8, 9].
3. The need for of systemic treatment is felt both with radiation when given in curative setting (only chemoradiation) and in adjuvant setting (chemoradiation postsurgical resection) in locally advanced tumors [10–12].

However the role of neoadjuvant chemotherapy (NACT) in locally advanced sinonasal malignancies is largely unaddressed. It is an interesting prospect considering anatomical proximity of sinonasal malignancies to vital structure and its locally aggressive behaviour both of which make gross total resection difficult. These tumors are responsive to chemotherapy in spite of the variable histologies seen at this site (esthesioneuroblastoma, sinonasal tumor of neuroendocrine differentiation, NUT midline tumors, and sinonasal tumor undifferentiated cancers).

Neoadjuvant chemotherapy before surgery may lead to regression of tumor and improvement in gross total resection rate in locally advanced tumors which in turn may improve the local control [7]. We routinely administer neoadjuvant chemotherapy in locally advanced resectable and borderline resectable sinonasal tumors with the aim of facilitating resection and improving local control. This audit was performed to study the efficacy (in terms of response rate), acute toxicity, and early outcomes with this strategy.

## 2. Methods

### 2.1. Selection of Cases

Sinonasal tumors are routinely discussed in a multidisciplinary clinic. Patients with the below mentioned criteria are referred for neoadjuvant chemotherapy before local treatment:

1. Locally advanced sinonasal tumors with extension of tumor beyond nasal and paranasal sinus:
  - Resectable: but resection would been morbid requiring extensive surgery and would have chances of incomplete gross total resection.
  - Unresectable: frank involvement of any vital structure or surgically inaccessible site making upfront surgery not possible.
2. ECOG PS 0–2.
3. Without distant metastasis.

### 2.2. NACT Delivery

These patients were treated with neoadjuvant chemotherapy. NACT consisted of cisplatin and etoposide. Cisplatin dose of 33 mg/m^2^ D1 to D3 and etoposide dose of 100 mg/m^2^ D1 to D3 were administered intravenously. Cisplatin was replaced with carboplatin (AUC-5 or 6) if the calculated serum creatinine clearance was below 60 mL/min. The chemotherapy was administered with standard premedications and antiemetic prophylaxis. Patients were given 1 liter of 0.9% NaCl hydration with magnesium and potassium supplementation from D1 to D3. Secondary prophylaxis with G-CSF was administered for patients having febrile neutropenia in C1. Two cycles of NACT were administered.

### 2.3. Treatment Post NACT

Following 2 cycles of NACT, patients were assessed with axial radiological imaging (either CECT or PET-CT). These patients were then discussed in skull base multidisciplinary clinic. Patients who had adequate response, which would facilitate gross total resection were offered surgical resection and adjuvant chemoradiation. Patients in whom, after 2 cycles, gross total resection was still not possible were offered radical chemoradiation. Patients who had progressed after NACT were considered for radical chemoradiation or palliative radiotherapy (RT) depending upon the patient's performance status and tumor volume. Palliative RT was delivered when tumor volumes were large and adequate tumoricidal RT doses could not be delivered without respecting the tolerance doses of nearby vital structures. These patients were followed up after treatment till death.

### 2.4. Data Collection

For this analysis, the data of these patients was acquired from a prospectively maintained head and neck cancer NACT database. Patients treated between August 2010 and August 2014 with sinonasal tumors and nonsquamous histology were selected. Data regarding baseline clinical details, staging, the indication of NACT, NACT details, response, adverse events, post-NACT local treatment details, pathological response, and outcome details were noted.

For this analysis, as NACT was given predominantly with the intention of having a gross total resection, the locoregional extent of tumor was charted. The charting was done with the following spaces being considered: involvement of cribriform plate, involvement of intracranial space with only extradural extension, involvement of intracranial space with intradural without involvement of brain, involvement of intracranial space with involvement of brain, involvement of orbit, and involvement of infratemporal fossa. This charting was done so that each of these space involvement would be analyzed as a factor predicting for achievement of resectability after NACT.

The response to NACT was noted in accordance with RECIST version 1.1. The adverse events during NACT were documented in accordance with CTCAE version 4.03. The pathological response rate was quantified as pathologic complete response (pCR) if no viable tumor was seen post-NACT and No-pCR if any viable tumor was seen post-NACT. The outcome data noted was progression free survival and overall survival. The progression free survival was calculated from date of start of NACT to date of progression (either locoregional or distant). Those patients who had not progressed were censored at their last follow-up. The overall survival was calculated from date of start of NACT to date of death. Those patients who had not died were censored at their last follow-up.

### 2.5. Statistical Analysis

Data was censored for analysis on September 30, 2015. Descriptive statistics was performed. Fisher's test was used to test whether there was a difference between response rate in the different histological subtypes considered. The analysis between achievement of resectability and different sites of involvement was also done by Fisher's test. The progression free survival and overall survival for each histology were computed by Kaplan Meier survival analysis. Log rank test was used for univariate analysis of PFS and OS. Cox regression analysis was used for multivariate analysis.

## 3. Results

### 3.1. Baseline Details

Twenty-five patients of sinonasal cavity cancer were identified. The baseline details are shown in Table 1. The median age of the whole cohort was 42 years (IQR 37–47 years). The ECOG PS was 0-1 in all 25 patients. There were 12 esthesioneuroblastoma patients and 13 SNEC patients. The Hyams grading of esthesioneuroblastoma was grade 2 in 1 patient, grade 3 in 8 patients, grade 4 in 2 patients, and not available in 2 patients. In these 2 patients, one patient's tissue was inadequate for grading while in other slides and blocks was not available for review. Out of 25 patients, nine patients had some form of previous local resections. Previous radiation exposure was seen in one patient while one of the patients had prior chemotherapy exposure. This patient had received 2 cycles of cisplatin and etoposide previously. Out of these 25 patients who all had locally advanced disease, 11 patients (44%) were considered unresectable and 14 patients were considered resectable upfront (56%).

### 3.2. Extent of Locoregional Spread and Reason for NACT

The extent of locoregional spread is shown in Table 2. All patient had skull base invasion. Regional lymph node involvement was seen in 08 patients (32%). Involvement of infratemporal fossa and parapharyngeal space was seen in 06 (24.0%) and 02 patients (08%), respectively.

The reason for NACT was dural involvement in 2 patients, brain parenchyma involvement in 4 patients, intracranial involvement in 5 patients, intracranial extension with orbital apex involvement in 2 patients, orbital involvement (extensive) in 1 patient, and infratemporal fossa involvement in 1 patient and extensive soft tissue disease in 10 patients.

### 3.3. NACT Compliance and Tolerability

Out of 25 patients 2 cycles of NACT were completed by all 25 patients. The median number of cycles delivered was 2 (IQR 2-3). Twelve patients (48%) received more than 2 cycles before locoregional treatment. The incidence of grade 3-4 toxicity in accordance with CTCAE version 4.03 was 76%. There was no grade 5 toxicity seen. The details of adverse events are shown in Table 3.

### 3.4. Response to NACT

The response was evaluable in all 25 patients after 2 cycles of NACT. The response was PR in 20 patients (80%, 95% CI 58.7%–92.4%), SD in 03 patients, and PD in 02 patients. The difference in response according to histological subtype is shown in Table 4. The response rate in esthesioneuroblastoma and in SN-NEC was 66.7% and 92.3%, respectively (*p* value = 0.160). The response rate in upfront resectable patients was 85.7% (12 patients out of 14) while it was 72.7% (08 patients out of 11) in upfront unresectable patients (*p* value = 0.623).

### 3.5. Post-NACT Resectability

Post-NACT 13 patients {*n* = 25, 52% (95% CI 33.5%–70.0%)} were resectable in the whole cohort of 25 patients. The achievement of resectability post-NACT with respect to the anatomical extent of the tumor is depicted in Table 5. Among the factors tested for achievement of resectability the resectability status before surgery had influence on achievement of resectability (Table 5). Resectability was achieved in 85.7% (12 out of 14) of patients who were considered resectable as opposed to 9.1% (1 out of 11) in patients who were considered unresectable (*p* = 0.136). Resectability was achieved in 60% (12 out of 20) of patients who responded to NACT as opposed to 20% (1 out of 5) in patients who did not respond to NACT (*p* = 0.136).

### 3.6. Post-NACT Treatment Details

Post-NACT 13 patients were resectable but 1 patient opted for CTRT. Post-NACT treatment received was surgery followed by adjuvant treatment in 11 patients, surgery without adjuvant in 1 patient, radical chemoradiation in 9 patients, and palliative RT in 2 patients. Two patients did not take local treatment after NACT. One patient had progressive disease after NACT and was not suitable for any local treatment. The other patient had near complete response and he did not want any further treatment.

Surgery was performed in 12 patients. The type of surgery done was craniofacial resection in 06 patients, craniofacial resection with medial maxillectomy in 01 patient, medial maxillectomy in 03 patients, sinonasal resection in 01 patient, and radical maxillectomy with orbital exenteration in 01 patient. It was a gross complete resection in all 12 patients. The pathological response was pathological complete response in 03 patients. All 12 patients were offered adjuvant treatment consisting of chemoradiation. However one patient declined adjuvant treatment as there was risk of vision loss associated with RT. 11 patients received adjuvant chemoradiation. Out of these 11 patients, 07 patients were treated with IMRT and 04 patients were treated with 3DCRT technique. The median dose to tumor bed (CTV) was 6000 cGy (IQR 6000-6000 cGy). All patients completed chemoradiation. The median number of weekly chemotherapy cycles (cisplatin 30 mg/m^2^) received were 6 (IQR 6-6).

Radical chemoradiation was done in 09 patients. Out of these, 07 patients were treated with IMRT and 02 patients were treated with 3DCRT technique. The median dose to tumor bed (CTV) was 6000 cGy (IQR 6000–6600 cGy). All patients except one completed radical chemoradiation. This patient progressed after 10# of RT and hence his RT was stopped. The median number of chemotherapy (cisplatin 30 mg/m^2^) cycles received were 5 (range 4–6).

Palliative RT was delivered by conventional method with the midline dose being 5500 cGy delivered in 22# in one patient and 5000 cGy in 25# in the other patient. Both patients had symptomatic relief postpalliative RT.

### 3.7. Outcomes

The median follow-up was 1.7 years (IQR 1.0–2.2 years). There were 6 patients who had progression. The first site of progression was local in 2 patients, local with distant in 1 patient, regional in 1 patient, and distant in 2 patients. The sites of distant failures were bony metastasis in 2 patients and regional lymph nodes in 1 patient. Five patients had died at the time of analysis and all deaths were due to disease progression.

The 2-year progression free survival and overall survival were 75% and 78.5%, respectively. The influence of different factors on PFS and OS can be seen in Table 6. The 2-year progression free survival was 91.7% and 57.0% in patients with esthesioneuroblastoma and SNEC, respectively. The stage of disease and response to chemotherapy was not found significantly associated with median PFS. However patients who had sufficient response for the disease to be considered as resectable had a 2-year PFS of 92.3% as opposed to 50.0% in patients who did not have sufficient regression of disease to make it resectable (*p* = 0.015). Patients who had unresectable disease upfront had a 2-year PFS of 45.5% as opposed to 100% in patients who were considered resectable upfront (*p* = 0.002). Among patients who underwent local treatment with radical intent patients undergoing surgery had better 2-year PFS than patients who received radical chemoradiation. The 2-year PFS in surgery group was 100.0% as opposed to 64.8% in patients treated with radical chemoradiation (*p* = 0.348).

**Figure 1:**
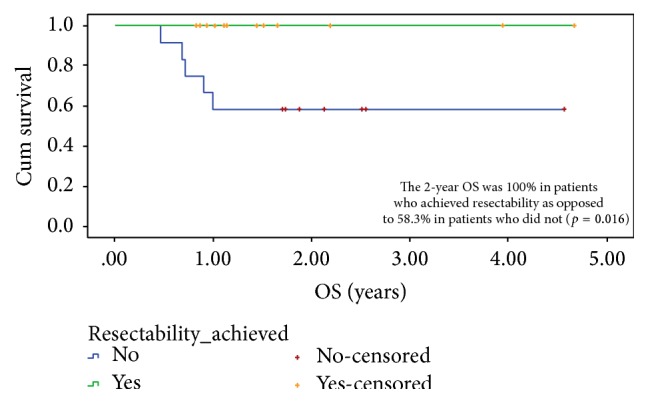
Two-year OS in accordance with achievement of resectability.

The 2-year OS (Figure 1) was 100% in patients who achieved resectability as opposed to 58.3% in patients who did not (*p* = 0.016). Similarly the 2-year OS was 100% in upfront resectable patients as opposed to 54.5% in unresectable patients (*p* = 0.008). Cox regression analysis failed to identify a single prognostic marker for PFS and OS. Both resectability achieved and upfront resectability status were considered for multivariate analysis.

## 4. Discussion

Sinonasal tumors have varied histology [1]. Squamous cell cancer histology seems to predominate [1]. We have already reported our results of locally advanced maxillary squamous cell cancers who were treated with neoadjuvant chemotherapy followed by local treatment [13]. In this analysis we wanted to focus on nonsquamous sinonasal malignancies. Locally advanced sinonasal malignancies have varied prognosis according to histology and stage [7]. In our previous report of sinonasal malignancies we had very poor outcomes with nearly 75% of our patients having a recurrence within 2 years [14, 15]. Since then, emphasis has been placed on use of neoadjuvant chemotherapy in patients with locally advanced and borderline resectable tumors as a strategy to improve outcomes.

All of these patients had Kadish C or D stage disease. The extent of the local tumor is highlighted in Table 2, where it can be noted that all patients had skull base invasion, half had orbital involvement, and one-third had regional lymph nodes involvement. These features are independently associated with inferior outcomes [16]. Previous reports suggest that these patients, when treated only with upfront chemoradiation, experience suboptimal outcomes. In a report from Chao, it was seen that patients treated with chemoradiation had 5-year local control of 51.2% versus a local control of 87.4% for patients undergoing surgery and RT [8]. In another retrospective analysis reported from New York, it was seen that in esthesioneuroblastoma radiotherapy alone was associated with an inferior outcome compared to surgical treatment [17]. On a similar note Gruber et al. also have recommended in their publication that irradiation alone, even when doses were escalated to 7300 cGy, is not sufficient in esthesioneuroblastoma [16]. All these reports probably carried an unintentional bias in a sense that in many instances it was the unresectable tumors which were subjected to radical chemoradiation. However, in view of the poor outcomes seen in our series and others with radical chemoradiation alone in unresectable tumors, we routinely consider these locally advanced patients for neoadjuvant chemotherapy to improve rates of local control and gross total resectability.

The neoadjuvant chemotherapy used here was cisplatin and etoposide. This is a standard regimen for treatment of high-grade neuroendocrine carcinomas. This regimen either alone or in combination with radiation in accordance with stage is used. Different combinations of chemotherapy have been used in the literature with platinum backbone. Many of these combinations have infusional 5 FU [18–21]. However we have logistic issues in delivering infusional 5 FU; this regimen is mainly used for squamous cell carcinoma and hence the above regimen was selected. The response rates achieved with this regimen are comparable to those reported in other series. The heartening fact is that the regimen was well tolerated, there was no mortality associated with this regimen, and the response rates achieved with this regimen in such advanced tumors was 80%. There is a small chance of progression of disease with NACT; it is a matter of concern but it helps in biologically selecting patients for further treatment.

Esthesioneuroblastoma was associated with high response rates and better outcomes than SNEC. This finding is similar to that reported in other studies [7]. The 2-year PFS and OS are much better now in our locally advanced and/or technically unresectable sinonasal cancers than they were seen in our previous report. This may be due to contribution of neoadjuvant chemotherapy along with better surgical and radiation techniques. Such tailored approaches in locally advanced sinonasal tumors with neoadjuvant chemotherapy have been associated with improvement in survival [22]. The importance of multidisciplinary approach seems a necessity in these tumors.

Interestingly the factors which impacted both PFS and OS were the resectability status of the patients prior to both surgery and after surgery. All of these patients had extensive disease but these patients were classified in multidisciplinary clinic upfront into potentially resectable or unresectable. Unresectable patients were those whose tumors had frank invasion of vital structures making them not a candidate for surgery. However the involvement in these tumors in most occasions either were encasing vital structures (like optic nerve, orbital apex) or had frank invasion of brain making these tumors an unlikely candidate for surgery even if they showed excellent response to NACT. As opposed to these patients termed resectable implied that gross total resection may not have been possible and if attempted would have required an extensive mutilating surgery. This classification had an impact on PFS and OS very similar to that of post-NACT resectability status. Our data validates this upfront classification as only 1 out of 11 inoperable patients was considered resectable after NACT as opposed to 12 out of 14 resectable patients. This data emphasizes proper selection criteria for selecting patients for NACT when it is administered to make the tumor resectable. However we failed to provide objective assessment criteria for such classification upfront. Upfront invasion of none of the anatomical landmarks considered in the study could predict achievement of resectability after NACT.

This study though retrospective has its own strengths and limitations. This is one of the few studies reporting on nonsquamous locally advanced sinonasal tumors who have been selected with homogenous criteria (Kadish C and D) and have been treated with homogenous protocols. The treatment planning of these cases had been done in a multidisciplinary clinic and post-NACT treatment was also decided in the same clinic. The limitation of this study is its retrospective nature and short follow-up. It is known in sinonasal tumors, especially esthesioneuroblastoma, to have delayed recurrence, in some instances even 10 years after initial treatment [7].

## 5. Conclusion

Sinonasal tumors are a group chemosensitive tumors. NACT with cisplatin and etoposide can achieve response rate of 80% in nonsquamous sinonasal tumors and is well tolerated. The protocol of NACT followed by local treatment is associated with improvement in outcomes.

## Figures and Tables

**Table 1:** Baseline details according to histological subtypes.

Variable	Esthesioneuroblastoma (*n* = 12 patients)	Sinonasal tumor with neuroendocrine differentiation (*n* = 13 patients)	Total (*n* = 25 patients)
Median age	40 years (IQR 36.5–42.75 years)	45 years (IQR 36.5–57.0 years)	42 years (IQR 37–47 years)
Gender			
Male	23	11	19
Female	04	02	06
ECOG PS			
PS 0-1	12	13	25
PS 2	00	00	00
Grade			
III-IV	10	13^*∗*^	08

^*∗*^All patients had high grade neuroendocrine tumors.

**Table 2:** Extent of locoregional spread.

Extent	Esthesioneuroblastoma (*n* = 12 patients)	SN-NEC (*n* = 13 patients)	Total (25 patients)
Involvement of cribriform plate	12 (100.0%)	13 (100.0%)	25 (100.0%)
Intracranial extension up to extradural region	06 (50.0%)	08 (61.5%)	14 (56%)
Intradural intracranial extension but brain parenchyma uninvolved	06 (50.0%)	04 (30.8%)	10 (40%)
Intradural extension with brain parenchyma involvement	02 (16.7%)	03 (23.1%)	05 (20%)
Involvement of orbit	06 (50.0%)	07 (53.8%)	13 (52%)
Involvement of infratemporal fossa	03 (25%)	03 (23.1%)	06 (24%)
Involvement of parapharyngeal space	01 (08.3%)	01 (7.7%)	02 (08%)
Involvement of regional lymph nodes	03 (25%)	05 (38.5%)	08 (32%)

**Table 3:** Adverse events in accordance with CTCAE version 4.03 observed during NACT. Numbers shown are actual patient numbers.

Toxicity	Grade 3	Grade 4
Anemia	02	00
Neutropenia	06	04
Thrombocytopenia	00	00
Febrile neutropenia	01	02
Nausea	00	00
Vomiting	00	00
Diarrhea	01	00
Increased serum creatinine	00	00
Transaminitis (raised SGOT/PT)	00	00
Hyponatremia	07	05
Hypokalemia	00	00
Hyperkalemia	00	00

**Table 4:** Response rates according to histological type.

	Esthesioneuroblastoma (*n* = 12 patients)	SN-NEC (*n* = 13 patients)	Total (25 patients)
CR + PR	8 (66.7%)	12 (92.3%)	20 (80%)
SD + PD	4 (33.3%)	01 (07.7%)	05 (20%)

**Table 5:** Factors affecting achievement of resectability.

	Presence of factor	Resectability achieved	*p* value on Fisher's test (one sided)	*p* value on binary logistic regression analysis
Anatomical factors				
Intracranial extension up to extradural region	Yes: 14	8	0.430	Not included
	No: 11	05		
Intradural intracranial extension but brain parenchyma uninvolved	Yes: 10	06	0.404	Not included
	No: 15	07		
Intradural extension with brain parenchyma involvement	Yes: 05	03	0.541	Not included
	No: 20	10		
Involvement of orbit	Yes: 13	07	0.582	Not included
	No: 12	06		
Involvement of infratemporal fossa	Yes: 06	02	0.281	0.851
	No: 19	11		
Involvement of parapharyngeal space	Yes: 02	01	0.741	Not included
	No: 23	12		
Surgical pre-NACT status	Unresectable: 11	01	0.000	0.003
	Resectable: 14	12		

Biological factors				
Pathology	E: 12	08	0.157	Not included
	SNE: 13	05		
Response	CR + PR: 20	12	0.136	0.139
	SD + PD: 05	01		

**Table 6:** The influence of different factors on PFS and OS.

Factor	Division	Median PFS in years	2-year PFS	*p* value (log rank test)
Histological type (*n* = 25)	Esthesioneuroblastoma	NR	91.7%	0.094
	SNEC	NR	57.0%	
Stage (*n* = 25)	Kadish C	NR	60.0%	0.347
	Kadish D	NR	81.4%	
Response (*n* = 25)	CR + PR	NR	77.3%	0.266
	SD + PD	NR	60.0%	
Resectability achieved (*n* = 25)	Yes	NR	92.3%	0.015
	No	1.10	50.0%	
Upfront status (*n* = 25)	Resectable	NR	100%	0.002
	Unresectable	1.10	45.5%	

Factor	Division	Median OS in years	2-year OS	*p* value (log rank test)

Histological type (*n* = 25)	Esthesioneuroblastoma	NR	91.7%	0.169
	SNEC	NR	64.5%	
Stage (*n* = 25)	Kadish C	NR	79.6%	0.677
	Kadish D	NR	75.0%	
Response (*n* = 25)	CR + PR	NR	82.7%	0.185
	SD + PD	NR	60.0%	
Resectability achieved (*n* = 25)	Yes	NR	100.0%	0.016
	No	NR	58.3%	
Upfront status (*n* = 25)	Resectable	NR	100%	0.008
	Unresectable	NR	54.5%	
